# Analysis of Psychological and Social Functioning in Undergraduate Students with a Specific Learning Disorder (SLD)

**DOI:** 10.3390/brainsci13071020

**Published:** 2023-06-30

**Authors:** Marika Bonuomo, Mara Marini, Nicoletta Vegni, Sergio Melogno, Giulia Torregiani, Stefano Livi, Gloria Di Filippo

**Affiliations:** 1 Faculty of Psychology, Niccolò Cusano University, 00166 Rome, Italy; nicoletta.vegni@unicusano.it (N.V.); sergio.melogno@unicusano.it (S.M.); gloria.difilippo@unicusano.it (G.D.F.); 2 Department of Social and Developmental Psychology, Sapienza University of Rome, 00185 Rome, Italy; mara.marini@uniroma1.it (M.M.); stefano.livi@uniroma1.it (S.L.)

**Keywords:** specific learning disorder, undergraduate students, psychological well-being, social well-being

## Abstract

It is considered to be particularly interesting to enrich the scientific overview investigating the comorbidities of specific learning disorders (SLDs) in young adults. Therefore, this study aims to investigate the psychosocial and relational profiles associated with the presence of learning difficulties in a population of university students. The hypothesis is that young adults with SLDs have lower psychological and socio-relational functioning than their typical-development peers. We further hypothesized that the socio-relational difficulties of students with SLDs could be explained not only by referring to the presence of a learning disorder, but also by considering some variables that may follow the experience of students with SLDs. The results highlighted that students with SLDs, compared to their typical-development peers, have low self-efficacy, high academic anxiety scores, emotional problems, and issues with peers. We finally suggest considering these aspects as early as the diagnostic process to facilitate an effective treatment plan for learning disorders to prevent, in terms of developmental trajectory, the manifestation of these aspects in adulthood.

## 1. Introduction

Several studies [1,2,3,4,5] have shown that specific learning disorders (SLDs) are often associated with socio-emotional difficulties. Musetti et al. [6] highlighted that academic difficulties associated with the experience of students with SLDs in childhood and adolescence are not exclusively reflected in the academic context but can also affect students’ socio-affective relationships. Several research studies support this perspective. For example, in a study on primary school students, Meadan and Hall [2] found that difficulties in academic information processing that are associated with a learning disorder were correlated with children’s difficulties in acquiring, processing, and discriminating social cues and rules. These results were also found in the work of Jarvis et al. [7], who found that adolescents and young adults with specific learning disorders appeared to be less able to interpret social situations than their typically developed peers. Furthermore, in a meta-analysis examining the impact of learning disorders on the social skills of school-age children, Kavale and Forness [8] found that 75% of students with SLDs showed a deficit in their social skills compared to the control sample. This is also consistent with Al-Yagon and Mikulincer [9], who indicated that primary school children with SLDs perceive lower levels of social acceptance than their no-SLD peers. A study by Estell [10], which examined the long-term effects of inclusion on children’s social behavior, showed while that primary school students with SLDs tend to be part of the social groups in the class, they consistently have lower social status compared to their peers. These differences have been persistent throughout the whole study pathway, suggesting that students with SLDs, while interacting within a social group for a long time, maintain difficulties in peer relationships.

Conversely, other studies aimed to understand the relationship between a specific learning disorder and students’ emotional and social difficulties have shown contradictory results [4,11,12,13]. A study on a group of Italian children designed to examine the relationship between SLDs, social relationships, self-esteem, and isolation, showed no significant differences in these dimensions between children and adolescents with and without SLD [1]. Thus, the presence of learning disabilities does not always appear to be associated with low levels of inclusion in the classroom. However, according to the authors, these results could be a consequence of the inclusive policies that characterize the Italian context. In fact, in the same study, it was found that children and adolescents with SLDs, who had attended a psychosocial education intervention, were more able to develop strategies to cope with emotional problems than the control group. Thus, it seems that the psychoeducational program promoted the development of greater self-awareness as an attitude of self-acceptance of oneself and one’s difficulties [1]. Social inclusion emerges as something that can be used to promote students’ adaptation and well-being, especially with a learning disorder, e.g., [14]. Despite these exceptions, research has shown that the low academic performance of students with SLDs can severely affect their self-esteem and social relationships, leading to genuine social rejection, e.g., [15,16,17]. However, in light of these contradictory results, research on this issue requires further investigation.

In contrast to the inconsistent findings of SLD-related interpersonal difficulties, the literature and clinical experience have demonstrated that learning disabilities can be a risk factor for psychological distress, increasing internalizing, anxious, and depressive symptoms [18]. The literature on this subject is extensive, particularly for adolescents and children. Many scientific studies have found a substantial comorbidity between learning difficulties and psychopathology [3,5,19,20]. Studies on children with SLDs found comorbidity rates of up to 60% [21,22]. For instance, the researchers discovered anxiety disorders in 28.8% of patients and mood disorders in 9.4% of them [22]. Visser et al. [5] also found relatively high rates of psychopathology in children with SLDs, especially when the disorder affects multiple domains (mixed SLD). In particular, the study by Willcutt and Pennington [23] examined the relationship between reading disabilities and internalizing and externalizing psychopathology in a large sample of twins with and without a reading disorder. The results showed that children and adolescents (8–18-years old) with reading difficulties were significantly more likely to be affected by psychopathological symptoms than the control group. This is easy to understand considering that children with specific learning disabilities face a unique set of emotional challenges due to their academic difficulties, e.g., [24], which can negatively impact behavior, emotion, and overall psychological well-being. In particular, academic anxiety tends to increase during childhood due to the central role of learning [25,26]; when learning difficulties are present, academic anxiety can represent a threat to school adaptation and success. In this regard, a comparative analysis conducted by Arnold et al. [27] found that adolescents with reading difficulties had higher levels of anxiety symptoms compared to the control group, with a higher prevalence for females. These difficulties seem to persist over time and are also present in university students with dyslexia, who experience higher levels of somatic complaints and depression [28]. In this regard, Chiappedi et al. [20], analyzing a group of students aged between 8 and 13, found that a positive school experience may reduce the risk of developing anxiety in adulthood. Therefore, poor accomplishment task performance appears to be quite important in predicting the presence of psychological issues, such as anxiety and depression [18]. This symptomatology might be aggravated at school by the inevitable comparison between one’s performance and that of one’s classmates [29,30]. Indeed, in this comparison, students with SLDs are frequently at a disadvantage compared to their peers. In this regard, many studies have shown that children with SLDs generally have a low overall perception of their abilities. Meltzer et al. [31] found that students with SLDs rated their abilities as average but had significantly lower perceptions of these than their peers. Furthermore, in a study conducted in primary school, Marinelli et al. [4] indicated that academic difficulties related to dyslexia can have an impact on self-esteem, besides academics [12,13,32] and other domains, especially for girls. The literature generally finds that primary school children with SLDs have a more negative self-concept than children without SLDs [20], influencing both school and interpersonal self-esteem [33] Ghisi et al. [28] report that this perceived inadequacy also persists in adulthood. Indeed, some studies report that university students with SLDs have significantly lower academic self-efficacy scores than their peers without SLDs, even when they demonstrate successful academic achievement [34,35]. Given the limited number of studies including college students with SLDs, we currently know little about the effects of low academic competence perceptions on well-being in the presence of a learning impairment, particularly among young adults.

All studies that were briefly examined above described the presence of a SLD as a risk factor for the psychological well-being of children and adolescents. These findings have piqued clinicians’ and researchers’ interest in the resilience abilities of children and adolescents with SLD. Resilience can be considered as an individual’s ability to develop personal resources to successfully face difficulties, challenges or adversities [36]. This ability can be useful in dealing with academic issues such as setbacks, and learning or relationship difficulties [37], which are common among children and youth with SLDs. According to Werner [38], resilience is a protective factor for the psychological well-being of children with a specific learning disability. However, Ghisi et al. [28] reported that university students with SLDs have high somatic complaints, social problems, attention problems, and depression when compared with peers with typical development, but they did not show a difference in resilience scores with respect to the control group. Also, in preadolescent children with SLDs, some studies found low levels of resilience [39]. Specifically, 75% of children and adolescents with SLDs had a low resilience index and high levels of depression and severe stress and anxiety.

To summarize, the psychological literature has found a robust link between learning difficulties and certain types of psychological discomfort in children and adolescents. Less evident is the relationship between the presence of the disease and relational functioning, as well as the role of resilience in the coping capacities of children and adolescents with SLDs. At the same time, research on the psychological and social profile of young adults with specific learning disorders is relatively limited compared to studies conducted with children and adolescents with SLDs, particularly about emotional-relational aspects [40]. Thus, to date, research examining factors associated with SLDs across the lifespan, especially in the context of higher education, has been limited. Considering the increasing number of students diagnosed with SLDs in higher education, this represents a gap in the relevant literature that needs to be addressed [40].

### Aims and Hypotheses

In the introductory phase of this study, the review of the psychological literature revealed several research gaps. To begin with, it appears essential to conduct more research on the psychological and social functioning of young adults with SLDs. Indeed, the majority of research has focused on the psychological effects of the disorder in children and adolescents, while university students’ experiences have been largely ignored. This is a limitation that must be surmounted, particularly in light of the increase in university students with SLDs, estimated at between 0.03% and 0.48% in Italian universities [41]. The numerous obstacles that the academic environment presents to all students, e.g., ref. [42] are especially significant for students with learning disabilities. Furthermore, the relational functioning associated with the presence of a SLD still appears to be unclear. This aspect necessitates further investigation due to the significance of social contexts throughout life. In actuality, the relational context of the students can serve as an irreplaceable source of social support and a resource for adaptation, e.g., [14,43], especially in situations of disadvantage. Therefore, it is necessary to comprehend the factors that may impede healthy interpersonal relationships among children, adolescents, and young adults with SLDs to design interventions aimed at enhancing this valuable protective factor for well-being.

Our study has two main objectives to address these gaps in the research. The first objective is to elucidate some aspects of psychological and social functioning among a sample of college students with SLDs. Particularly, psychological functioning has been defined from two perspectives. On the one hand, it has been considered as the level of adaptation attained in the academic context. Indeed, educational experiences in children, adolescents, and young adults can be valuable predictors of personal well-being. The studies described in the introduction to our work, as well as in the section dedicated to describing the measures used in our study, showed that this component of psychological functioning in children and adolescents with SLDs can be particularly challenging. Psychological functioning, on the other hand, was considered as the degree of psychological distress. Numerous studies agree on the occurrence of psychological problems (e.g., anxiety and depression) in the presence of learning disorders. In terms of social functioning, we looked at the quality of peer relationships as an indication of well-being. These factors were taken into account because the literature has shown, despite inconsistent findings, that children and adolescents with SLDs have relational difficulties. However, this issue needs to be addressed more effectively with young adult populations. Consequently, following the presented literature (e.g., [1,2,3,4,5,6,21,22,28,33,34,35,44]), we hypothesized that young adults with SLDs, like children and adolescents, have lower psychological and socio-relational functioning than their peers with typical neurological development (Hypothesis 1). The second objective is to define factors that may hinder relationships between young adults with SLDs. Indeed, the literature identifies significant relational difficulties in young people with learning disabilities, e.g., [1,2,3,4,5,6]. However, in this studies’ field, results are inconclusive, and research has only focused on child and adolescent populations, e.g., [4,11,12,13]. The absence of clear relationships between the presence of the disorder and the social functioning of children, adolescents, and adults with SLDs suggests that the quality of interpersonal relationships among students with SLDs is likely influenced by a variety of factors. In our study, we hypothesized that the socio-relational difficulties of students with SLDs could be explained not only by referring to the presence of learning disorders, but by considering the psychological functioning and variables that may follow the experience of students with SLDs (Hypothesis 2). Nevertheless, our hypotheses are based on the findings of studies primarily conducted with children and adolescents. Consequently, they were developed based on what we know so far about the functioning profile of groups of students of different developmental ages from those of interest to the current study. As a result, while it is conceivable to postulate that the psychological-social functioning of young adults follows a trajectory comparable to that of younger students based on the limited research available, our hypotheses are mostly exploratory.

To achieve the goals of the study, we recruited a group of students with a diagnosis of a SLD and another group of students whose development was considered to be typical. To examine the psychological and social well-being of adolescents and young adults from a range of viewpoints, we applied a battery of clinical instruments that are commonly employed to assess their psychological functioning.

## 2. Materials and Methods

### 2.1. Participants and Procedure

44 undergraduate students recruited from an Italian university (mean age = 26.98, SD = 8.45, minimum age = 19, maximum age = 53) were enrolled for the study. The clinical sample was collected with the collaboration of the university’s inclusion office, which sent invitations to students to join the study. The invitation was accepted by 17 students (mean age = 27, standard deviation = 8.96, and 58.8% females) with a learning disorder diagnosed and certified by territorial services. As for the diagnosis, two students claimed a reading disorder, one claimed a calculation disorder, and the remaining fifteen claimed a mixed learning disorder. Snowball sampling was used to recruit the control group from the same university. 27 students with typical development (mean age = 26.96, SD = 8.29, 55.5% females) responded to the survey. The questionnaire was administered through a Google Forms link. The study was authorized by the university’s ethics committee. Before completion, participants were informed of the aims and methods of the study and provided informed consent to participate in the research.

### 2.2. Measures

To assess the psychological and social functioning of young adults with SLD, we investigated their adaptation through measures validated in the Italian context and widely used in the clinical setting to evaluate adolescents’ and young adults’ psychological–social well-being.

#### 2.2.1. Psychological Adaptation

Students’ psychological adaptation was assessed using several AMOS tests (study skills and motivation: assessment and orientation tests for secondary school and university) [45] and the Symptom Checklist-90-R by Derogatis [46].

The AMOS questionnaire evaluates study skills, cognitive styles, and emotional and motivational learning components, as well as the advantages and disadvantages of different study methods. Specifically, the Beliefs Questionnaire (in Italian: Questionario sulle convinzioni-QC) and the Anxiety and Resilience Questionnaire (in Italian: Questionario sull’ansia e la resilienza-QAR) were used. 

For the QC, 8 items were used to assess implicit theories of intelligence, i.e., the extent by which students perceive their intelligence as permanent and invariable (4 items; α = 0.93) or modifiable through learning and experience (4 items; α = 0.87). The concept of implicit theories of intelligence was applied in educational research to describe how students perceive their ability to improve their skills [47].These concepts were developed from the Mindset Theory [48], according to which students can have a growth or fixed mindset about their intelligence. Research on these topics has shown that these theories can influence students’ psychological and behavioral functioning as they create belief systems that guide how they see themselves and the world [49]. Growth-minded students believe their intelligence is malleable; they perceive challenges as opportunities for improvement, do not give up on obstacles, and are strongly motivated to learn [48]. In contrast, fixed-minded students believe their intelligence is unchanging; consequently, they react negatively to failure, are unable to regulate their effort to achieve a task or persist in the event of difficulty, and have low expectations of success [50]. There has recently been a growing interest in studying implicit theories of intelligence in students with learning disabilities [47,51]. Indeed, during their academic experiences, these students may encounter obstacles or difficulties which, in addition to promoting the belief that their competence is low, can reduce the perceived control over their results, self-confidence, motivation, and persistence in academic tasks. In the AMOS test, students answered questions about their implicit theories of intelligence using a 6-point Likert scale to indicate their level of agreement with each item (from 1 = strongly disagree to 6 = strongly agree). 

In addition, the QC included five items measuring self-perceived academic competence (α = 0.87), to which students responded using a 5-point Likert-type scale (from 1 = very low ability to 5 = exceptionally high ability). Extensive research has been conducted on perceived academic competencies [52]. Regardless of the theoretical perspectives employed, the perception of competence is a “fundamental psychological need that has pervasive effects on daily life, cognition, and behavior” [53]. Such perceptions are heavily influenced by social context and school-based social comparison processes. In this regard, studies on the Big-Fish-Little-Pond-Effect [30] have concentrated on the effects of classmates’ abilities on the development of self-perceived academic competence (academic self-concept). The BFLPE is an appropriate resource for comprehending the experiences of disadvantaged students who may be less capable than their peers in certain academic disciplines [54]. For students with SLDs, learning disabilities may harm their academic self-concept and self-image, particularly when compared to those of their high-performing peers. This negative self-concept, in addition to harming the psychological health of these students, can also hinder their performance and school motivation [52].

On the other hand, the QAR comprises 14 items and identifies anxiety related to school distress (7 items; α = 0.93) and resilience attitudes (7 items; α = 0.59). Negative emotional states associated with school discomfort are risk factors for the psychosocial and academic well-being of children and adolescents with learning disabilities, e.g., [55,56]. Many conditions in the school setting may facilitate these negative experiences e.g., [57], for example, the scholastic difficulties due to the characteristics of the learning disability. Furthermore, environments that are not welcoming and responsive to the needs of children and adolescents with learning difficulties can aggravate the distress e.g., [58]. Research on risk factors for the well-being of children and adolescents with SLDs has fueled a growing interest in the construct of resilience, described as a dynamic process in which risk and protective factors interact and allow for the emergence of adaptive resources [59,60]. Therefore, psychosocial distress is not an inevitable fate for children and adolescents with SLDs. Instead, research has demonstrated the presence of various resilience factors, which, in the presence of some risk factors linked to the presence of an SLD, can explain the success in various areas of life of children and adolescents with learning disabilities [61]. However, research needs further study on this topic as the results are still unclear, especially about young adults with SLDs [28,39]. Students responded to each item using a 5-point Likert-type scale (from 1 = strongly disagree to 5 = strongly agree).

The Symptom Checklist-90-R by Derogatis [46] was used to assess psychological distress. The SCL-90-R is a self-administered tool widely used in clinical and research settings. The scale is composed of 90 items and assesses a broad spectrum of psychological problems and psychopathological symptoms, ranging from internalizing symptoms to externalizing symptoms. Specifically, the SCL-90-R includes the following subdimensions [46]: somatization (discomfort attributable to a perceivable bodily dysfunction); obsession–compulsion (thoughts, impulses, and actions that are experienced as persistent and irresistible, egodystonic, or undesirable); interpersonal sensitivity (feelings of inadequacy and inferiority); depression (feelings of hopelessness and other cognitive and somatic depression correlates); anxiety (general symptoms of anxiety, including nervousness, tension, and panic attacks); hostility (thoughts, feelings, and actions indicative of a negative affective state of anger); phobic anxiety (persistent specific fear reactions deemed irrational or disproportionate); paranoid ideation (paranoid thinking characterized by, among other things, suspicion, grandeur, and delusions); and psychoticism (symptoms indicative of withdrawal and isolation, up to and including the first-rate symptoms of schizophrenia). The measure is used for the evaluation of stress levels and psychopathological indices in children and adolescents with SLDs and in their parents e.g., [33]). In our study, for each item, the students were asked to respond using a 0 to 4 scale to indicate the frequency of the presented symptomatology (from 0 = not at all to 4 = extremely often). Each dimension’s reliability was adequate: somatization (α = 0.91), obsession–compulsion (α = 0.87), interpersonal sensitivity (α = 0.83), depression (α = 0.92), anxiety (α = 0.90), hostility (α = 0.82), phobic anxiety (α = 0.81), paranoid ideation (α = 0.77), and psychoticism (α = 0.85).

#### 2.2.2. Socio-Emotional and Behavioral Adaptation

The Strengths and Difficulties Questionnaire (SDQ) by Goodman in its Italian adaptation by De Giacomo et al. [62] was used to assess behavioral and emotional difficulties. The SDQ questionnaire consists of 25 items and allows for the exploration of students’ prosocial behavior and students’ difficulties or strengths in five macro-areas: emotional problems, behavioral problems, hyperactivity and inattention, problem relationships with peers, and prosocial behaviors. The SDQ is frequently used as a screening instrument for behavioral and emotional problems in children and adolescents [63,64,65]. In addition, it is utilized in educational research and clinical practice to investigate the functioning of children and adolescents with SLDs, e.g., [66]. Several studies based on this instrument found that children with SLDs are at a higher risk than typically developing children for developing emotional and behavioral difficulties. Multiple factors and experiences contribute to these adaptation difficulties [67]. Particularly, studies on the socioemotional dimensions of children with SLDs have revealed that during school social activities, children with learning difficulties can experience high levels of distress. Concerning behavioral issues, studies involving children with SLDs have revealed that these children exhibit high levels of hyperactivity, conduct problems, aggressiveness, and antisocial behaviors, e.g., [67,68]. Nonetheless, these issues appear to be the indirect result of learning disorders. The number and severity of behavioral and emotional problems among students are substantially affected by the child’s and environment’s reactions to an SLD diagnosis. Participants answered each item on a 3-point scale (0 = false, 1 = partly true, 2 = true). According to the scoring guide [62], the scores were then recorded. Specifically, a 3-point scale was used for both positive (0 = false, 1 = partially true, 2 = true) and negative (2 = false, 1 = partially true, 0 = true) items. The number of scores was calculated according to the indicated macro ranges (minimum score = 0, maximum score = 40).

### 2.3. Data Analysis

SPSS Statistics27 [69] for Windows was used to analyze the collected data. Before analyzing the data, skewness and kurtosis were examined, and variables were standardized. All variable distributions were determined to be within normal limits [70].

The procedures for data analysis were conducted in two phases. In the first phase, we created a dummy variable (grouping variable) to distinguish between the two participant groups: students with typical development (grouping variable = 0) and students with SLDs (grouping variable = 1). Following that, psychological and emotional–behavioral adaptations were compared between the two groups using an analysis of covariance (ANCOVA). In particular, the mean scores for the following dimensions were compared between the two groups of participants: implicit theories of intelligence, self-perceived academic competence, academic anxiety, resilience, psychological distress (somatization, obsession–compulsion, interpersonal sensitivity, depression, anxiety, hostility, phobic anxiety, paranoid ideation, psychoticism), emotional problems, behavioral problems, hyperactivity/inattention, problematic peer relationships, and prosocial behavior. In each ANCOVA model, differences between groups were accounted for by gender and age.

In the second phase, based on the results of the ANCOVA models, a hierarchical multiple regression model was evaluated. The variance inflation factor (VIF) and tolerance statistics were used to ascertain multicollinearity, and the Durbin–Watson test [38] was utilized to evaluate autocorrelation issues. As a criterion variable, the regression model included the problematic peer relationships. One of the aims of the study was to ascertain which factors may hinder social functioning in young adults with SLDs. As predictors of problematic peer relationships, the grouping variable (students with SLDs and students with typical development), gender, and age were included in the first block of the regression model. In the second phase, predictors to be included in the regression model were chosen based on the areas of psychological functioning identified as deficient in students with SLDs during previous ANCOVA analyses. In particular, self-perceived academic competence, academic anxiety, and emotional difficulties were added as predictors in the second block. As stated in the previous section, the hypothesis underlying this study is that the socio-relational difficulties of students with SLDs can be understood not only by referring to the presence of the disorder, but also by considering some variables that may result from their school and life experiences.

## 3. Results

Below are the ANCOVA results for each of the considered variables. Students’ gender and age were included as covariates in all analyses. The descriptive statistics for each dimension analyzed are presented in Table 1.

Concerning QC covariance analyses (ANCOVAs) revealed no statistically significant differences between groups’ implicit theories of intelligence, both for fixed (*F*_(1,40)_ = 1.11, *p* = 0.30, *η_p_*^2^ = 0.03) and malleable conceptions (*F*_(1,40)_ = 0.19, *p* = 0.67, *η_p_*^2^ = 0.01). In contrast, statistically significant differences emerged between groups in terms of self-perceived academic competence (*F*_(1,40)_ = 7.89, *p* = 0.008, *η_p_*^2^ = 0.17). Specifically, students with SLDs had lower mean scores in self-perceived academic competence (*M* = 3.35, *DS* = 0.8, *N* = 17) than typically developing students (*M* = 3.84, *DS* = 0.43, *N* = 27). 

Concerning the QAR results, covariance analyses (ANCOVAs) revealed statistically significant differences between the groups in mean academic anxiety scores (*F*_(1,40)_ = 4.75, *p* = 0.03, *η_p_*^2^ = 0.11). Specifically, the students with SLDs (*M* = 3.44, *DS* = 0.87, *N* = 17) exhibited greater academic anxiety than the students with typical development (*M* = 2.77, *DS* = 1.05, *N* = 27). In contrast, there were no statistically significant differences between groups regarding resilience (*F*_(1,40)_ = 0.41, *p* = 0.52, *η_p_*^2^ = 0.01).

Concerning psychological distress measures, covariance analyses (ANCOVAs) revealed no statistically significant differences between groups on all dimensions examined: somatization (*F*_(1,40)_ = 1.69, *p* = 0.20, *η_p_*^2^ = 0.04); obsession–compulsion (*F*_(1,40)_ = 1.47, *p* = 0.23, *η_p_*^2^ = 0.03); interpersonal sensitivity (*F*_(1,40)_ = 1.62, *p* = 0.21, *η_p_*^2^ = 0.04); depression (*F*_(1,40)_ = 0.76, *p* = 0.39, *η_p_*^2^ = 0.02); anxiety (*F*_(1,40)_ = 1.45, *p* = 0.24, *η_p_*^2^ = 0.03); hostility (*F*_(1,40)_ = 0.763, *p* = 0.39, *η_p_*^2^ = 0.02); phobic anxiety (*F*_(1,40)_ = 0.65, *p* = 0.43, *η_p_*^2^ = 0.02); paranoid ideation (*F*_(1,40)_ = 1.84, *p* = 0.18, *η_p_*^2^ = 0.04); and psychoticism (*F*_(1,40)_ = 1.95, *p* = 0.66, *η_p_*^2^ = 0.01). However, the results revealed gender differences in the scores on the following dimensions: somatization, obsession–compulsion, depression, and anxiety. Then, an analysis of variance (ANOVA) with a 2 (clinical group/control group) × 2 (female/male) factorial design was conducted to test the presence of interactions between groups on the considered distress dimensions. Only a main effect of gender was observed for somatization (*F*_(1,40)_ = 6.75, *p* = 0.01, *η_p_*^2^ = 0.14), depression (*F*_(1,40)_ = 10.01, *p* < 0.01, *η_p_*^2^ = 0.03), and anxiety (*F*_(1,40)_ = 6.73, *p* = 0.01, *η_p_*^2^ = 0.14). Specifically, females (somatization: *M* = 1.01, *DS* = 0.82, *N* = 25; depression: *M* = 1.27, *DS* = 0.88, *N* = 25; anxiety: *M* = 0.95, *DS* = 0.77, *N* = 25) had higher mean scores on all dimensions than males (somatization: *M* = 0.45, *DS* = 0.51, *N* = 19; depression: *M* = 0.58, *DS* = 0.57, *N* = 19; anxiety: *M* = 0.46, *DS* = 0.44, *N* = 19).

Concerning behavioral and emotional difficulties, covariance analyses (ANCOVAs) were performed to investigate differences between students with SLDs and students with typical development, controlled for age and gender, on the following dimensions: emotional problems, behavioral problems, hyperactivity and inattention, problematic peer relationships, and prosocial behavior. Only the students’ scores on emotional problems (*F*_(1,40)_ = 4.41, *p* = 0.04, *η_p_*^2^ = 0.10) and problematic relationships with peers (*F*_(1,40)_ = 9.16, *p* < 0.01, *η_p_*^2^ = 0.19) revealed statistically significant differences between groups. In particular, students with SLDs reported higher scores than students with typical development for both emotional problems and problematic peer relationships (clinical group: *M* = 3.59, *DS* = 2.21, *N* = 17; control group: *M* = 1.78, *DS* = 1.67, *N* = 27). Concerning emotional problems, the results revealed gender differences (*F*_(1,40)_ = 11.68, *p* < 0.01, *η_p_*^2^ = 0.23): females (*M* = 4.64, *DS* = 2.60, *N* = 25) showed higher scores than males (*M* = 2.37, *DS* = 1.80, *N* = 19). To investigate the presence of interactions between groups in emotional problems, an analysis of variance (ANOVA) with a 2 (clinical group/control group) × 2 (female/male) factorial design was conducted. The findings confirm a gender main effect (*F*_(1,40)_ = 11.68, *p* < 0.01, *η_p_*^2^ = 0.23), but also show a significant interaction effect (*F*_(1,40)_ = 4.23, *p* = 0.05, *η_p_*^2^ = 0.10). The interaction effect demonstrated that the association between gender and emotional problems differed between groups (students with SLDs and students with typical development). Emotional problems did not differ between males with SLDs (*M* = 2.29, *DS* = 0.80, *N* = 7) and males with typical development (*M* = 2.41, *DS* = 0.61, *N* = 12) (mean difference = 0.13, *p* = 0.99); however, females with SLDs (*M* = 6.20, *DS* = 0.67, *N* = 10) showed higher scores in emotional problems than typically developing females (*M* = 3.60, *DS* = 0.55, *N* = 15) (mean difference = −3.91, *p* < 0.01).

In conclusion, the results revealed statistically significant differences between students with a learning disability and typical developing students on the following dimensions: self-perceived academic competence, academic anxiety, and emotional and peer relationship problems. It seems that the presence of a learning disorder is a factor that hinders adaptive functioning in several areas. Our first hypothesis was therefore confirmed.

On the basis of these preliminary findings, a two-stage hierarchical multiple regression analysis was performed to elucidate the factors that may hinder peer relationships among young adults with SLDs. Before conducting regression analyses, correlation analyses between the variables under consideration were carried out (Table 2).

Correlation analyses revealed a negative and statistically significant association between the grouping variable (students with SLDs and students with typical development) and self-perceived academic competence (*r* = −0.37, *p* = 0.01). In addition, positive and significant correlations were observed between the grouping variable (students with SLDs and students with typical development) and academic anxiety (*r* = 0.32, *p* = 0.03) and between the grouping variable (students with SLDs and students with typical development) and peer relationship problems (*r* = 0.43, *p* < 0.01). This suggested that the presence of an SLD was associated with a low perception of academic competence, high academic anxiety, and difficulties with peer relationships. On the other hand, no significant correlations were observed between the grouping variable (students with SLDs and students with typical development) and emotional problems. Regarding self-perceived academic competence, significant and negative correlations emerged with academic anxiety (*r* = −0.054, *p* < 0.001), emotional problems (*r* = −0.33, *p* = 0.03), and peer relationship problems (*r* = −0.45, *p* < 0.01). This indicated that as students’ sense of academic competence increases, academic anxiety, emotional problems, and relationship difficulties decrease, and vice versa. Concerning academic anxiety, positive and significant correlations emerged with emotional problems (*r* = 0.50, *p* < 0.01) and peer relationship problems (*r* = 0.34, *p* = 0.02). This suggested that as academic anxiety levels rise, so did emotional and relationship problems, and vice versa. Finally, there was a positive and significant correlation between emotional problems and peer relationship difficulties (*r* = 0.51, *p* < 0.01): the more difficult it was to manage emotional components, the more troublesome relationships with peers became, and vice versa.

The hierarchical multiple regression results are presented in Table 3. 

In the model, difficulties in peer relationships were inserted as the criterion variable. The presence or absence of SLDs, self-perceived academic competence, academic anxiety, and emotional problems were included as predictors. 

Before the analyses, tolerance and variance inflation factor (VIF) values were computed to determine the presence of multicollinearity among the independent variables. The outcomes demonstrated the absence of multicollinearity (tolerance values ranging between 0.50 and 1; VIF values ranging between 1 and 2.02) [39]. The Durbin–-Watson coefficient was used to test residual autocorrelation. In the regression model, the Durbin–Watson value was adequate (1.60).

In the first step of the regression model, the grouping variable (students with SLDs and students with typical development), age, and gender were entered as predictors (Model 1 in Table 3); this allowed for an examination of the relationship between the presence of an SLD and relational difficulties, controlled for gender and age. In the second step (Model 2 in Table 3), self-perceived academic competence, academic anxiety, and emotional problems were entered as additional predictors. Indeed, we wanted to understand whether these variables could add explained variance to the previous model. This procedure allowed us to examine whether the presence of an SLD was still a predictor of students’ relational difficulties even after accounting for psychological functioning.

Hierarchical multiple regression analyses revealed a significant relationship between the grouping variable (students with SLDs and students with typical development) and peer relationship problems (*β* = 0.43; *p* < 0.01), which, as discussed in the previously presented analyses, were more prevalent among students with SLDs than among students with typical development. There were no significant associations between age and gender and peer relationship problems. The variables entered in the first block explained 14.5% of the variance in the criterion variable (*F*_(3,40)_ = 3.44, *p* = 0.03). In the second step, when self-perceived academic competence, academic anxiety, and emotional problems were added to the regression equation, the change in *R*^2^ was statistically significant (*F_change_* _(3,37)_ = 5.45, *p* < 0.01), and the model explained 35.2% of the variance (*F*_(6,37)_ = 4.89, *p* < 0.01). Specifically, the association between beliefs in academic abilities and peer relationship problems was negative and statistically significant (*β* = −0.36; *p* = 0.05). In contrast, the association between emotional problems and peer relationship problems was positive and statistically significant (*β* = 0.38; *p* = 0.02). There was no association between academic anxiety and peer relationship problems. Fascinatingly, after adding self-perceived academic competence, academic anxiety, and emotional problems to the model in the second step, the grouping variable (students with SLDs and students with typical development) was no longer significantly associated with the criterion variable. Our second hypothesis is supported by these results. 

The findings of the regression analyses indicated that the presence of an SLD can hinder the formation of positive relationships with peers. However, when different aspects of students’ psychological functioning are considered, the presence of an SLD was unrelated to relational dynamics. This enables us to consider that the presence of an SLD has no direct relationship with peer relationship problems. To comprehend the social functioning of students with SLDs, it seems essential to examine their self-perceived academic competence and emotional regulation abilities. Positive self-perception, in particular, appears to be a protective factor for social functioning. On the contrary, a poor ability to manage one’s emotions might be a risk factor in the interpersonal domain.

## 4. Discussion

Specific learning disorders (SLDs) are often associated with psychological and socio-emotional difficulties [2,3,5,7,8,9,10,19,20,21]. The learning difficulties of students with SLDs are not only reflected in the academic context but can also affect everyday social relationships [6,71]. In particular, the poor academic performance of students with SLDs may lead to a more negative self-concept than their typically developed peers [72] and undermine their self-esteem [4,44] and overall perception of their abilities [12,13,31,32]. The literature also shows that children with SLDs generally have difficulties in social relationships, sometimes leading to outright social rejection [9,17]. In particular, they report difficulties in processing social signs and rules [2,8,10]. There is also scientific evidence of high comorbidity between learning disorders and psychopathology [3,5,19,20]. SLD children may present higher levels of academic anxiety symptoms [25,26], which can hinder adaptation and academic success [43]. However, resilience can be a useful trait in responding to academic difficulties and relational difficulties and supporting psychological well-being in children with specific learning disorders [19,20]. Nevertheless, low levels of resilience have been found in SLD students [39].

It is important to highlight that most of the scientific evidence on these aspects refers to a sample under the age of 18. For this reason, we intended to investigate these aspects in a sample of university adults to broaden the literature of the scientific landscape, as well as to verify their developmental continuity or discontinuity. There are few studies investigating these aspects [7,34,35]. 

The present study investigated the students’ psychological–social functioning in a population of young university adults. The results confirmed the presence of some differences in psychological and social functioning between students with SLDs and typically developing students. In particular, students with SLDs seem to have more problems in the following areas: self-perceived academic competence, academic anxiety, and emotional problems.

Regarding self-perceived academic competence, our study demonstrated an association between SLDs and low perceptions of academic abilities. These results corroborate findings from several studies involving children and adolescents [4,12,13,32]. Thus, young adults are characterized by a lower perception of their abilities than peers without SLDs. This is in line with several studies that have reported how college students with SLDs have lower scores than their peers without SLDs in academic self-efficacy, even in the presence of a successful academic pathway [34,35,73]. Furthermore, as stated in the literature, this study confirmed high academic anxiety scores in young adults at university, as well as those observed in the literature with children and adolescents [44]. Regarding emotional difficulties, it is interesting to note that in our sample of university adults with SLDs, high difficulties emerged, both in terms of symptoms and management, in comparison with participants of the control group, especially for girls. This evidence is consistent with previous studies where more emotional symptoms were found in girls than in boys [4,23,27,73]. 

In this study, we also investigated the variables potentially implicated in the relational difficulties of students with SLDs. Several authors emphasize that the difficulties that accompany the experience of students with SLDs are not limited to the academic setting but can also affect their social relationships in their everyday lives e.g., [6,14]. However, there are contradictory results regarding this issue. This evidence can be comprehended by considering various facets of the psychological functioning of SLD students. In our study, it was confirmed that students with SLDs report more peer problems than students without SLDs. However, when we considered other areas of functioning—for example, cognitive (perceptions of academic competence) and emotional (emotional problems) functioning—the presence of a SLD was no longer related to relational difficulties with peers. These findings permit us to reflect on several issues. To begin with, having a learning disorder is not necessarily a condition that hinders interpersonal relationships. Instead, it is necessary to pay attention to the psychological consequences of an SLD diagnosis. Indeed, there are numerous ways in which learning disorders can compromise the psychosocial well-being of students. For instance, low academic performance, which is frequently associated with a diagnosis of a SLD, may exacerbate negative outcomes of social comparison processes [29,74] and threaten students’ evaluations of various dimensions of the self, including perceptions of academic competence [75]. The literature has abundantly demonstrated that such perceptions play a significant role in developmental trajectories. According to our findings, the relationship between academic competence and relational difficulties is negative and significant, and hence this perception can be regarded as a protective factor for social well-being. Similarly, a poor capacity to regulate emotions might be a predictor of interpersonal problems. In our study, emotional issues were found to be positively and strongly connected with relationship problems and, hence, to be a risk factor for social adaptation.

Therefore, our findings provide insights for the design of research and interventions intended to promote the psychological and social well-being of students. Although SLD should be considered a risk factor for adjustment in various domains, it appears necessary to pay attention to the multidimensional nature of students’ experiences and consider the various facets of psychological functioning. These findings partially explain the discordant results that have emerged from research on the relational difficulties of students with SLDs. To comprehend the experience of these students, it seems essential to consider not only the presence of a learning disorder, but also how this may impact cognitive and emotional responses. In the literature, these aspects have been largely ignored, particularly in population of young adults. An in-depth investigation revealed that these socio-relational difficulties were only indirectly connected to the disorder. The perceived academic competence and emotional problems of students with SLDs explained their relationship difficulties.

## 5. Limitations and Future Directions

Our study has several limitations that must be considered. Firstly, the design of the study was cross-sectional. This allowed us to observe the relationships between the variables of interest. However, as socio-relational difficulties emerge in interaction with the academic path, it might be particularly revealing in future research to implement longitudinal designs [76]. Secondly, the study’s sample size was small. While we tried our best to avoid the impact of possible selection biases, the small size of the sample is in itself a factor limiting the generalizability of the findings. In particular, future studies including larger and more heterogeneous samples of SLD patients would allow for exploring the interactions between the variables investigated to determine whether psychological functioning could be a mediator of the direct association between a SLD and interpersonal functioning. A bigger sample size would also allow for the use of more advanced statistical models, such as structural equation models. Thirdly, although the measures employed in this study are widely used in clinical settings to assess student well-being, future research could also employ measures based on theoretical models successfully applied in social–psychological research. Marsh’s model might be used, for instance, to measure perceptions of academic competence [77]. This would allow for the identification of the multidimensional nature of the academic self-concept regarding perceptions of competence in the mathematical and verbal domains. These subdimensions might then be investigated along with the type of diagnosed disorder. Fourthly, the investigation was entirely conducted quantitatively. For future research, it may be advantageous to implement qualitative–quantitative research using a mixed-methods approach that can integrate the strengths of both study perspectives. This could give voice to the students’ experiences and shed light on the mechanisms and processes at work in their adaptation. Finally, the study simply evaluated individual student performance, without considering the role of environmental circumstances. Future studies may benefit from investigating the function of ecological systems in the individual’s life experiences and devising studies that take into account the ongoing interaction between humans and the environment.

## 6. Implications

Our research has several implications. The results of our study corroborate what has already emerged in research conducted so far with children and adolescents with SLDs and confirm the presence of differences in psychological and social functioning between students with SLDs and students with typical development. In particular, the most impaired areas were the following: self-perceived academic competence, academic anxiety, and emotional problems. This suggests that, from a clinical perspective, attention needs to be paid to the psychological consequences of a SLD diagnosis and the repercussions of these across the lifespan. The instruments used in our study could therefore be administered by specialized and properly trained professionals, such as learning tutors, to detect any deficit areas early on. This would make it possible to prevent forms of distress, such as negative self-perception or emotional problems, that might persist into adulthood. Concerning academic institutions, our study offers useful pointers to inclusion services for activities to monitor the college experience of students with SLDs. Indeed, activities could be proposed to enhance the perceptions of academic competence of students with SLDs, while also taking into account the possible negative outcomes associated with academic anxiety. In addition, during the diagnostic update provided in the transition between school cycles [78], the clinician could pay attention to the areas of psychological functioning that emerged as deficient in students with SLDs in our study. In addition, our research has shown that the presence of a SLD may be a risk factor for students’ social adjustment. However, students’ perceptions of academic competence and emotional regulation skills seem to be better predictors of interpersonal problems. Therefore, these aspects should be given special consideration by the educational community to create a positive school experience for all students, and particularly for students with SLDs, who frequently exhibit problematic interpersonal relationships, e.g., [7,8,9,10]. Thus, developing clinical interventions aimed at improving young adults’ emotion regulation skills and self-efficacy seems to be a way forward to improve their interpersonal relationships and, consequently, their social well-being.

## 7. Conclusions

Despite various limitations, this study contributes to our understanding of psychological and social functioning in undergraduate students with SLDs. Students with SLDs exhibited low perceived academic competence, academic anxiety, and emotional and interpersonal difficulties. An in-depth investigation revealed that these socio-relational difficulties were only indirectly connected to the disorder. The perceived academic competence and emotional problems of students with SLDs explained their relationship difficulties. Thus, it appears that these aspects of SLD students’ psychological and social functioning should be the focus of early intervention. To promote an effective treatment plan for learning disabilities, it is proposed that these factors should be considered at the moment of the diagnostic process, using evaluation instruments for emotional, social, and relational issues. This may prove beneficial for preventing the manifestation of socio-relational difficulties in adulthood.

## Figures and Tables

**Table 1:** Descriptive analyses.

					Students with SLDs		Students without SLDs	
	Min.	Max.	Mean (all samples)	SD	Mean	SD	Mean	SD
Theories of intelligence (fixed)	1.25	6	3.1	1.3	3.32	1.37	2.90	1.18
Theories of intelligence (malleable)	1	5.5	2.9	1.1	3.03	1.18	2.88	1
Self-perceived academic competence	1.60	4.8	3.7	0.6	3.35	0.81	3.84	0.43
Academic anxiety	1	5	3	1	3.44	0.88	2.77	1.05
Resilience	2.14	5	3.5	0.6	3.45	0.61	3.57	0.59
Somatization	0	2.8	0.8	0.8	0.96	0.80	0.65	0.71
Obsession–Compulsion	0	3.5	1.2	0.8	1.43	0.68	1.13	0.85
Interpersonal sensitivity	0	2.3	0.8	0.7	0.95	0.68	0.69	0.64
Depression	0	3.4	1	0.8	1.11	0.76	0.88	0.88
Anxiety	0	2.8	0.7	0.7	0.89	0.71	0.64	0.65
Hostility	0	3	0.65	0.68	0.76	0.82	0.57	0.57
Phobic anxiety	0	1.9	0.3	0.5	0.37	0.51	0.24	0.50
Paranoid ideation	0	3	0.9	0.7	1.12	0.91	0.81	0.61
Psychoticism	0	2.3	0.6	0.6	0.62	0.64	0.53	0.61
Emotional problems	0	10	3.7	2.5	4.6	2.96	3.1	2.1
Behavioral problems	0	6	2.2	1.4	2.5	1.59	2	1.2
Hyperactivity/Inattention	0	7	2.7	1.9	2.9	1.89	2.5	1.9
Problematic peer relationships	0	8	2.5	2.1	3.59	2.21	1.78	1.67
Prosocial behavior	5	10	8.9	1.3	8.65	1.69	9	1.04

**Table 2:** Correlation analyses.

	1	2	3	4	5	6	7
1. SLD	--						
2. Gender	0.03	--					
3. Age	0.00	0.33 *	--				
4. Self-perceived academic competence	−0.37 *	0.00	−0.34 *	--			
5. Academic anxiety	0.32 *	0.31 *	0.14	−0.54 **	--		
6. Emotional problems	0.29	0.45 **	0.04	−0.33 *	0.50 **	--	
7. Problematic peer relationships	0.43 **	0.07	−0.10	−0.45 **	0.34 *	0.51 **	--

* The correlation is significant at the 0.05 level (two-tailed). ** The correlation is significant at the 0.01 level (two-sided).

**Table 3:** Hierarchical multiple regression results.

Predictor	Beta	*p*-Value	Tolerance	VIF
Model 1 (*R*^2^_*adj*_ = 0.145; *F*_(3,40)_ = 3.43, *p* = 0.03)				
SLD	0.427 **	0.004	0.10	1.001
Gender	0.101	0.503	0.89	1.126
Age	−0.139	−0.928	0.89	1.124
Model 2 (*R*^2^_adj_ = 0.352; *F*_(6,37)_ = 4.89, *p* < 0.001)				
SLD	0.206	0.140	0.807	1.239
Gender	−0.009	0.955	0.575	1.738
Age	−0.229	0.133	0.679	1.472
Self-perceived academic competence	−0.362 *	0.045	0.495	2.022
Academic anxiety	−0.070	0.674	0.549	1.820
Emotional problems	0.379 *	0.024	0.579	1.727

* *p* ≤ 0.05; ** *p* ≤ 0.01; difficulties in peer relationships were the criterion variable.

## Data Availability

The data presented in this study are available on request from the corresponding author. The data are not publicly available since data sharing is not in accordance with the consent provided by participants on the use of data.
